# Synthesis of Uniformly Sized Bi_0.5_Sb_1.5_Te_3.0_ Nanoparticles via Mechanochemical Process and Wet-Milling for Reduced Thermal Conductivity

**DOI:** 10.3390/ma14030536

**Published:** 2021-01-22

**Authors:** Bo-In Park, Miri Shin, Jaeho Park, Jae-Seung Lee, Seung Yong Lee, Seunggun Yu

**Affiliations:** 1 Department of Materials Science and Engineering, Korea Advanced Institute of Science and Technology (KAIST), Daejeon 34141, Korea; boin0905@gmail.com; 2 Materials Architecturing Research Center, Korea Institute of Science and Technology (KIST), Seoul 02792, Korea; T20233@kist.re.kr; 3 Department of Materials Science and Engineering, Korea University, Seoul 02841, Korea; jslee79@korea.ac.kr; 4 Department of Materials Science and Engineering, Seoul National University, Seoul 08826, Korea; haha0728@kist.re.kr; 5 Insulation Materials Research Center, Korea Electrotechnology Research Institute (KERI), Changwon 51543, Korea

**Keywords:** BST, nanoparticles, thermal conductivity, mechanochemical process, wet-milling

## Abstract

In this study, Bi_0.5_Sb_1.5_Te_3.0_ (BST) nanoparticles (NPs) with high crystallinities were synthesized via a mechanochemical process (MCP). X-ray diffraction (XRD), and Raman and X-ray photoelectron spectroscopy (XPS) spectra of the BST NPs showed that the Bi, Sb, and Te powders successfully formed BiSbTe phase and transmission electron microscopy (TEM) images, verifying the high crystallinity and smaller size, albeit agglomerated. The as-synthesized BST NPs with agglomerated clusters were ground into smaller sizes of approximately 41.8 nm with uniform distribution through a simple wet-milling process during 7 days. The thermal conduction behaviors of bulk alloys fabricated by spark plasma sintering (SPS) of the BST NPs were studied by comparing those of samples fabricated from as-synthesized BST NPs and a BST ingot. The thermal conductivities (*κ*) of the BST nanocomposites were significantly reduced by introducing BST NPs with smaller grain sizes and finer distributions in the temperature range from 300 to 500 K. The BST nanocomposites fabricated from wet-milled BST NPs offered ultralow *κ* values of 0.84 W m^−1^ K^−1^ at approximately 398 K.

## 1. Introduction

Over the past few decades, thermoelectric (TE) materials, which can directly convert heat into electricity based on the Seebeck effect, have received significant attention because they possess the ability to harvest waste heat, and can serve as useful energy resources [1,2,3]. The TE modules integrated with TE materials have been widely employed not only in a variety of refrigerators for cooling, but also in sustainable power systems for energy generation [4,5,6]. The conversion efficiencies of TE materials have been evaluated using the dimensionless figure of merit, *ZT*, where *ZT* = (*S*^2^*σT κ*^−1^), and *S*, *σ*, and *T* are the Seebeck coefficient, electrical conductivity, and absolute temperature, respectively, while *κ* is the thermal conductivity, which is divided into electronic (*κ_e_*) and lattice (*κ_l_*) terms [7]. From this equation, it is predicted that the TE performance can be enhanced by increasing the power factor (*S^2^σ*) or decreasing *κ*. In the viewpoint of *κ*, *κ_l_* is caused by phonons, also referred to as lattice vibrations [8]. The phonons are intensively scattered at the nanostructured interfaces in the given materials, which induces a significant reduction in *κ_l_*, while *κ_e_* is preserved, thereby resulting in improved *ZT* values of the TE materials [9,10,11]. Recent efforts have focused on introducing nanostructures, such as nanoparticles (NPs), nanowires (NWs), nanoporous media, and nanocomposites, which are easily capable of forming numerous grain boundaries, into bulk TE materials [12,13,14,15,16,17]. Among a variety of routes for introducing nanostructures into bulk TE materials, the mechanochemical process (MCP) is an effective approach to obtain alloy NPs from elemental precursors by self-ignition and propagation reaction during high-energy ball milling under dry conditions [18,19,20,21,22,23]. Thus, this method is advantageous for designing TE materials because of the facile fabrication process that facilitates large-scale TE NP production, wherein the NPs serve to provide smaller grain sizes and larger grain boundaries for lower *κ_l_* values.

Among the various types of TE materials, the BiSbTe ternary alloy, which is a solid solution constructed by alloying Bi_2_Te_3_ and Bi_2_Se_3_, is the most promising TE semiconductor because of its high ZT value near room temperature, making it useful for practical TE applications [24,25,26]. By optimizing its composition with Bi_x_Sb_2−x_Te_3_, the *p*-type bulk alloy fabricated from the NPs by ball milling and spark plasma sintering (SPS) exhibited the highest *ZT* value of 1.4 at approximately 373 K, which ushered in a new avenue for versatile low-temperature TE cooling modules [9]. In particular, the high *ZT* value was obtained by introducing nanostructures into the TE material, while controlling the sizes and qualities of the BiSbTe NPs with an average size of approximately 20 nm.

Here, we further studied Bi_0.5_Sb_1.5_Te_3.0_ (BST) TE materials, which were synthesized by means of the MCP, followed by post-wet-milling, to obtain their crystalline NPs with smaller sizes and uniform distribution. The crystallographic characteristics of the BST NPs were analyzed. Furthermore, the *p*-type bulk alloy of BST was fabricated via SPS, and its *κ* with temperature was evaluated and compared to that of the BST alloy from an ingot.

## 2. Experimental

### 2.1. Synthesis of BST NPs

The BST NPs were synthesized without any additives using a planetary-ball-mill machine (Pulvertisette 5, FRITSCH GmbH, Idar-Oberstein, Germany). Bi (99.999%, Alfa Aesar, Haverhill, MA, USA), Sb (99.98%, Sigma Aldrich, St. Louis, MS, USA), and Te (99.99%, Sigma Aldrich, St. Louis, MS, USA) powders were mixed at a predetermined ratio as a function of the BST molar ratio. The powder mixture (~10 g in total) was mixed in a round-ended stainless-steel jar (80 mL in volume) with ZrO_2_ balls (50 g of 5–10 mm diameter). The mild wet-milling process was performed for 7 days in a Nalgene bottle (150 mL) containing BST powder (4 g), anhydrous ethanol, and ZrO_2_ balls with a diameter of 1 mm, followed by drying in a vacuum oven to obtain powder.

### 2.2. Preparation of BST Nanocomposites

The BST nanocomposite was prepared using an SPS machine (SPS-825, Fuji electronics, Tokyo, Japan) at 450 °C and a heating rate of 50 °C min^−1^ under a load of 40 MPa for 10 min to yield a cylindrical bulk sample with a diameter of 12.7 mm for measuring thermal diffusivity and specific heat values.

### 2.3. Characterization

The crystallinity and phase purity of as-synthesized BST NPs were determined via X-ray diffraction (XRD, P1, Bruker, Billerica, MA, USA) at a scan rate of 0.5 °/s with Cu K*α* radiation (*λ* = 1.54 Å) and Raman spectroscopy (LabRAM ARAMIS, Horiba, Kyoto, Japan) with an Ar-ion laser (*λ* = 514.5 nm) as the excitation source (beam spot diameter = 1 μm), where the Raman scattered light signal was collected in a backscattering geometry using the × 50 microscope objective lens. The core states were analyzed by means of X-ray photoelectron spectroscopy (XPS, PHI 5000, ULVAC-PHI, Kanagawa, Japan) with a monochromator (Al K*α* source of 1486.6 eV with a C 1s peak of 286.6 eV), and the beam size was 100 × 100 μm^2^ with a detection limit of 0.5 at% under a base pressure of 2.0 × 10^−7^ Pa. The crystallographic characteristics of as-synthesized BST NPs and morphologies of as-milled BST NPs were analyzed via transmission electron microscopy (TEM, TitanTM 80-300, FEI, Hillsboro, OR, USA) combined with energy-dispersive spectroscopy (EDS) (XFlash, Bruker, Billerica, MA, USA), and scanning electron microscopy (SEM, Inspect F50, FEI, Hillsboro, OR, USA). The size distribution of the BST NPs was obtained by analyzing the high-resolution SEM images using the software ImageJ (http://rsb.info.nih.gov/nih-image/). The temperature-dependent *κ* of the BST bulk alloy samples was calculated from the equation *κ* = *T_d_* × *C_p_* × *ρ*, where the *T_d_*, *C_p_*, and *ρ* are the thermal diffusivity (mm^2^ s^−1^), specific heat (J g^−1^ K^−1^), and density (g cm^−3^), respectively. The thermal diffusivity and specific heat values were measured using a laser flash apparatus (LFA447, Netzsch, Selb, Germany) with a xenon flash lamp source from 300 to 500 K, and all the measurements were performed 5 times after stabilizing each temperature for accuracy. The specific heat was obtained through a temperature increase of the top surface of the sample and calibration using a pyro-ceramic reference sample with a known specific heat. The density values of the bulk samples were measured using the Archimedes method.

## 3. Results and Discussion

### 3.1. Synthesis of Crystal-Pure, Uniformly Sized BST NPs

**Figure 1:**
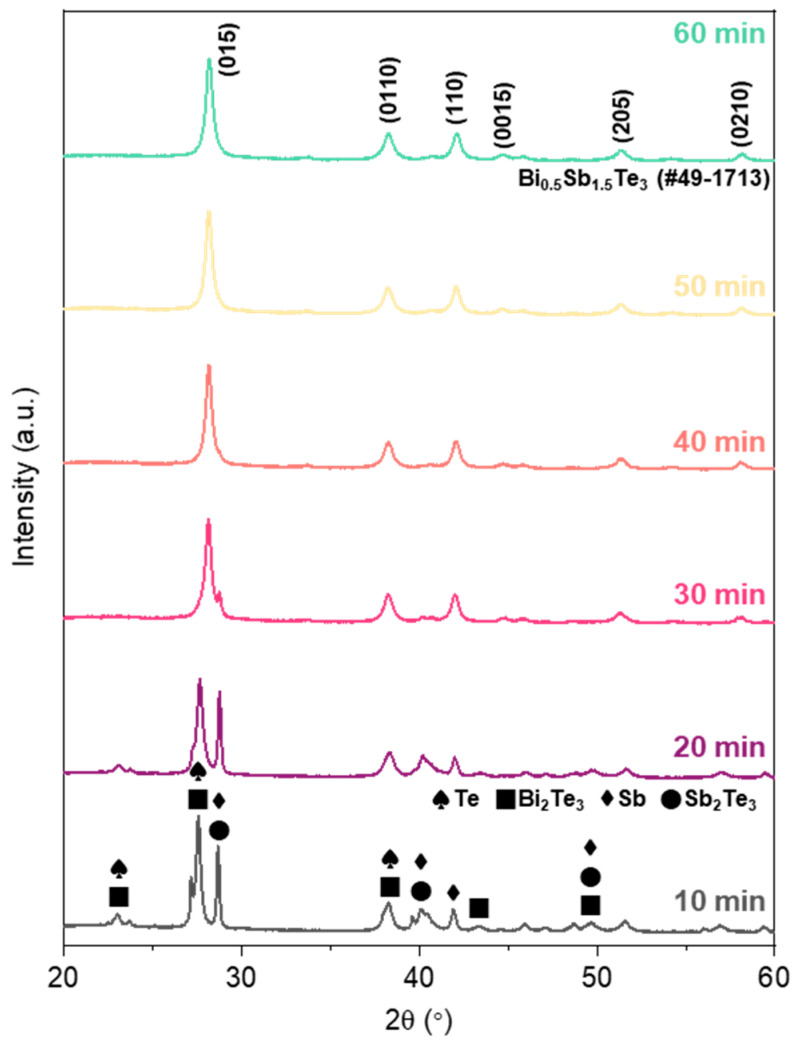
X-ray diffraction (XRD) spectra of Bi_0.5_Sb_1.5_Te_3.0_ (BST) nanoparticles (NPs) as a function of mechanochemical process (MCP) time.

The phase evolution of the BST NPs synthesized through the MCP was evaluated using XRD according to the reaction time, as illustrated in Figure 1. When the reaction time reached approximately 10 min, peaks of Bi_2_Te_3_ and Sb_2_Te_3_ appeared, while the peaks of elemental Te and Sb still remained. After 30 min, the peaks of pure elements disappeared, and the characteristic peak of BST was suddenly revealed because the MCP accompanied an abrupt phase change during the formation of the compound [27]. Therefore, we can conclude that the synthesis of BST was accomplished from the formation of Bi_2_Te_3_ and Sb_2_Te_3_, followed by combining these compounds into a single phase of Bi_0.5_Sb_1.5_Te_3_ with the increase in the MCP reaction time. After 40 min, the phase-pure BST NPs were apparently synthesized, wherein each peak was consistent with the standard BST (JCPDS 49-1713). According to Scherrer’s equation, the primary crystallite sizes of BST NPs were estimated to be 14.4 and 13.5 nm for the MCP times of 40 and 60 min, which were calculated from the full-widths at half-maximum (FWHM) of 0.58 and 0.63, respectively. It seems that only the particle size was decreased as the MCP time was increased from 40 to 60 min without phase evolution. That is, the MCP process facilitated a simple yet rapid synthesis of BST NPs within tens of minutes by the explosive process through self-propagating reaction, while common routes such as the solvothermal process and sol–gel method for the synthesis of BST NPs required elaborate conditions and a gradual reaction time [28,29,30].

**Figure 2:**
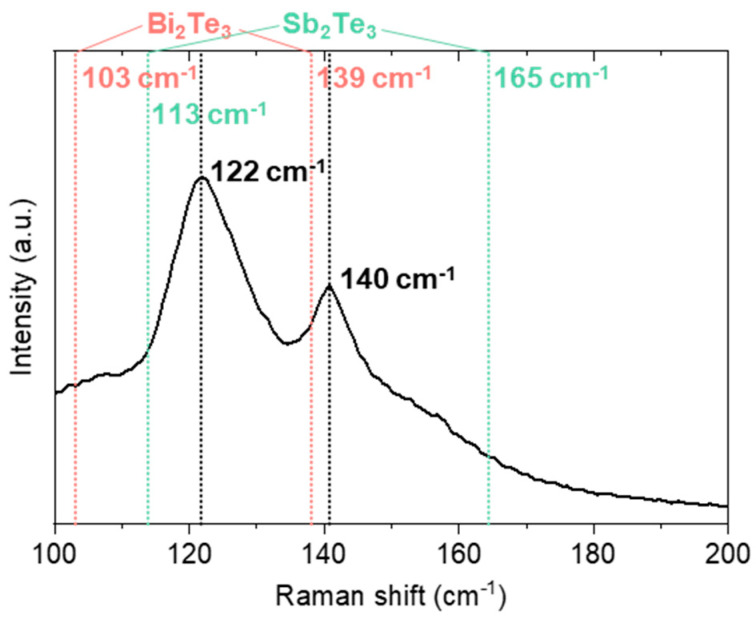
Raman spectra of the as-synthesized BST NPs.

To observe the phase purity of BST NPs synthesized by MCP, Raman scattering analysis was further performed, because the multicomponent compounds with complex composition sometimes form the secondary phases. The common BST peaks of E_g_^2^ (122 cm^−1^) and A_1g_^2^ (140 cm^−1^) observed in the Raman spectra indicated that the BST synthesized via MCP had a homogeneous stoichiometric composition between those of Bi_2_Sb_3_ and Sb_2_Te_3_, as shown in Figure 2 [31].

**Figure 3:**
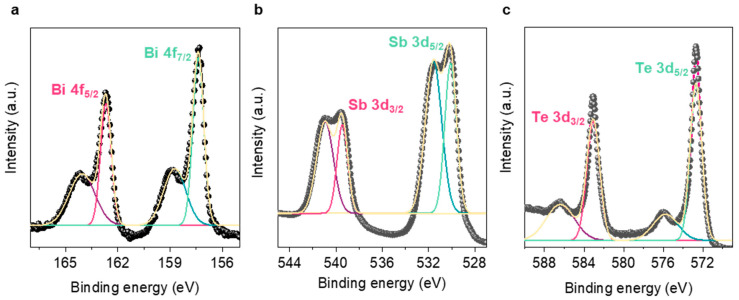
High-resolution X-ray photoelectron spectroscopy (XPS) spectra of (**a**) Bi 4f, (**b**) Sb 3d, and (**c**) Te 3d orbitals.

Figure 3 shows the states of the core levels on the surface of the as-synthesized BST powders. Bi 4f_7/2_, _5/2_ peaks were located at 157.3 and 162.7 eV, Sb 3d_5/2_, _3/2_ were evident at 530.1 and 539.5 eV, and Te 3d_5/2_, _3/2_ were observed at approximately 572.7 and 583.0 eV [32]. The signals in the other higher-energy regions were peaks exhibited by oxidation states and were expected to be slightly oxidized during the sample (pellet form) fabrication process.

**Figure 4:**
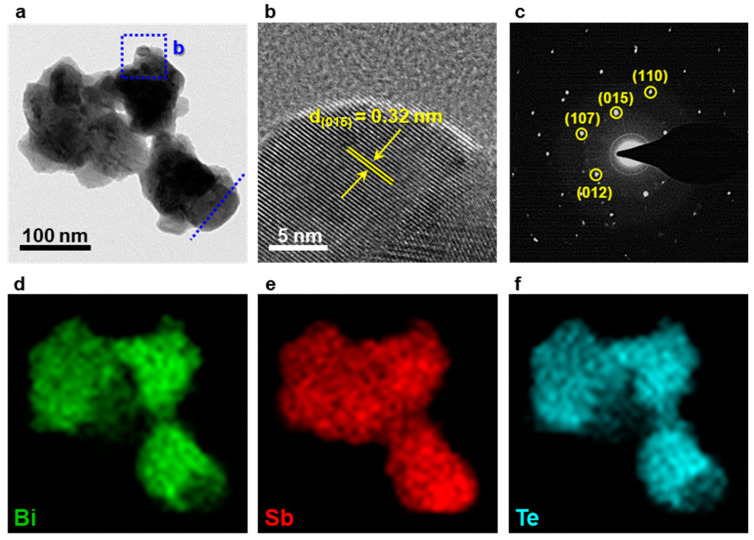
(**a**,**b**) High-resolution (HR)-transmission electron microscopy (TEM) images, (**c**) selected-area electron diffraction (SAED) pattern, (**d**–**f**) elemental mapping images from STEM-EDS.

The high-resolution (HR)-transmission electron microscopy (TEM) image shows that the BST particles had a certain crystallinity, although they were agglomerated with a size of several hundred nanometers, as presented in Figure 4a,b. The d-spacing value of 0.32 nm corresponded to the (015) plane of the BST crystal and had an interplanar distance identical to its stoichiometry. The patterns of the selected-area electron diffraction (SAED) in the (110), (015), (107), and (012) planes were consistent with the XRD patterns, as displayed in Figure 4c (Figure 1). Notably, no trace of oxidation on the surface of the particles was observed by TEM analysis. However, as shown in XPS spectra, as-synthesized BST NPs were likely oxidized to form an oxide layer because the mechanochemical process was not carried out under a controlled oxygen-free atmosphere. In addition, the elemental mapping images obtained via EDS showed that the elements—Bi, Sb, and Te—were entirely distributed throughout the material, which was also confirmed from the line-scan profile, as shown in Figure 4d–f, respectively (Figure S1). The elemental composition in weight and atomic ratio of BST NPs calculated by SEM-EDS were almost identical to the stoichiometric ratio of Bi_0.5_Sb_1.5_Te_3.0_, in which [Bi]/[Bi] + [Sb] = 0.31 and [Te]/[matrix] = 1.42, as shown in Table 1 (Figure S2).

**Figure 5:**
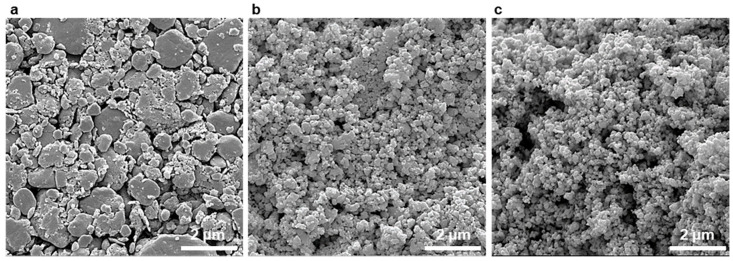
SEM images of (**a**) the as-synthesized BST NPs and post-wet-milled BST NPs for (**b**) 3 and (**c**) 7 days.

Although the MCP is beneficial for simultaneously synthesizing and pulverizing the alloy composed of primary smaller particles, as-fabricated alloy particles are likely to easily be agglomerated due to the dry process. Thus, it has been commonly used to apply the post-wet-milling process using solvent to yield the NPs with homogeneous size distribution in wide applications such as solar cells, water-splitting, and Li-ion batteries [33,34,35,36]. In this sense, a simple wet-milling process was introduced to disperse the agglomerates of the as-synthesized BST NPs, as well as to produce smaller and more uniformly distributed particles. The as-synthesized BST NPs exhibited an agglomerated form with a wide nano- to micron-scale distribution, as shown in Figure 5a. The size of the BST NPs evidently decreased by several hundred nanometers after wet-milling for 3 days, and further decreased by a few dozen nanometers after 7 days, as shown in Figure 5b,c.

**Figure 6:**
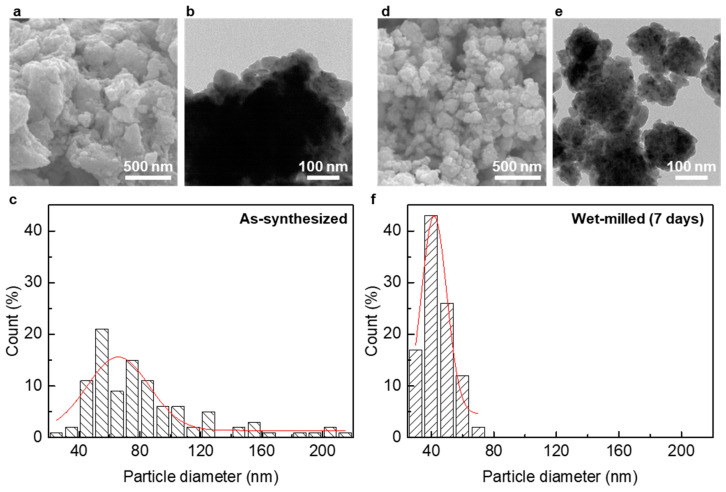
(**a**,**d**) SEM, (**b**,**e**) TEM, and (**c**,**f**) plots of size distribution of the as-synthesized BST NPs and post-wet-milled BST NPs, respectively.

To verify the smaller and more uniformly distributed BST NPs via wet-milling, we also investigated the statistical size distributions of BST NPs below 250 nm by analyzing the high-magnification SEM images. As-synthesized BST NPs formed the large-sized clusters comprising the NPs with a variety of size distributions, as shown in Figure 6a (Figure S3a). From the TEM image, each particle was hardly observed due to their agglomerates with huge sizes, as shown in Figure 6b (Figure S3b). For the BST NPs under 250 nm excluding those micron-sized, the as-synthesized BST NPs exhibited a wide distribution in size, in which the average value obtained from its gauss curve was approximately 65.7 nm, as shown in Figure 6c. Meanwhile, the post-wet-milling process during 7 days enabled the further pulverization of BST NPs, resulting in a smaller size and finer distribution, as shown in Figure 6d (Figure S3c). In addition, the TEM image of the wet-milled BST NPs showed that the particles were split into smaller clusters under approximately 100 nm, in which each particle with smaller sizes was distinctively observed, as shown in Figure 6e (Figure S3d). The size distribution of the wet-milled BST NPs moved to a smaller level with a narrow distribution, as shown in Figure 6f. In particular, the size of wet-milled BST NPs was totally reduced to below 80 nm, and the average value was approximately 41.8 nm, in which their crystallite size was also obviously reduced from 15.9 to 9.0 nm after wet-milling (Figure S4). Thus, the wet-milled BST NPs had a uniform size distribution, which could contribute to the control of the thermal conduction property by providing originally nano-sized grains with finer distribution for grain boundary engineering.

### 3.2. Thermal Conduction Properties of Bulk BST Nanocomposites

**Figure 7:**
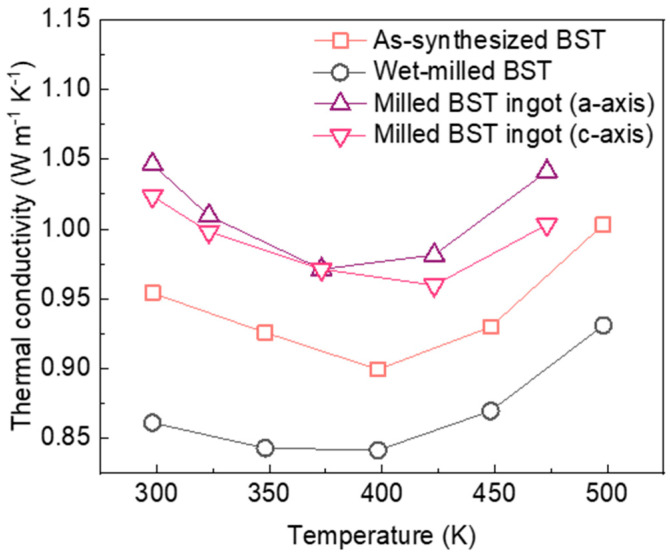
Temperature-dependent *κ* of the as-synthesized BST, post-wet-milled BST, and the BST ingot as the axes.

To prepare the bulk TE alloy sample, as-synthesized and as-milled BST NPs were poured into a graphite mold, followed by SPS. For comparison, the BST sample fabricated from the ingot (BST ingot) was prepared along its geometric axis. The nanostructured bulk alloy of the BST NPs fabricated via SPS may be isotropic based on the random orientation of the grains [37]. The total *κ* of the as-synthesized BST NPs, as-milled BST NPs, and BST ingot samples were analyzed with respect to temperature, as shown in Figure 7. The whole samples exhibited common trends in *κ* whereby the value decreased and then increased with temperature due to bipolar diffusion [37]. The BST ingot samples exhibited an axis-dependent *κ*, in which the value in the c-axis (perpendicular to pressing direction) was lower than that in the a-axis direction due to their anisotropic growth during sintering. The *κ* value of the as-synthesized BST nanocomposite was less than that of the BST ingot regardless of the axes. After wet milling, the *κ* value was significantly reduced and became inferior to that of the as-synthesized BST nanocomposite over the entire temperature range. At room temperature, the κ value of the BST nanocomposite was approximately 0.86 W m^−1^ K^−1^, while the lowest *κ* value of the nanostructured BST was approximately 0.84 W m^−1^ K^−1^ at approximately 398 K, which were estimated to be 82.2% and 86.6% of those of the BST ingot, respectively. This was attributed to the strong phonon scattering at grain boundaries and suppression of the minority carrier mobility in the nanostructured BST with smaller grain sizes and finer distributions than those of the initial case, while the pulverization of ingots through mechanical milling occasionally yielded large-sized particles above the sub-micrometer range with inhomogeneity in size distribution, resulting in the formation of large domains in the bulk alloy [37,38]. It was also noteworthy that the as-milled BST NPs are advantageous for their highly dense packing capable of reducing pores because of their uniform size distribution. Therefore, the post-wet-milling process of the mechanochemically synthesized BST NPs is promising for potential TE materials by controlling the *κ* value.

## 4. Conclusions

Here, BST was successfully synthesized via MCP using high-energy ball milling. The approach yielded BST NPs with high crystallinities without the formation of a secondary phase, but this approach agglomerated NP clusters with a large size distribution. By introducing the mild wet-milling process, the agglomerated clusters were split into NPs with smaller sizes and more uniform distributions than those of the initial case. The BST nanocomposites fabricated from the mechanochemically synthesized NPs exhibited lower κ values compared to those of the BST ingot over the entire temperature range. In particular, the reduction in *κ* was significant after performing wet-milling on the NPs relative to the as-synthesized NPs because of the smaller grain sizes with uniformity of the wet-milled NPs. Consequently, our two-step process, MCP, and wet-milling entail an performance improvement for potential TE applications by reducing the *κ* value.

## Figures and Tables

**Table 1:** Compositions of as-synthesized BST NPs estimated by EDS.

Element	Additive Content (phr%)	TTI (s)
Bi	20.0	13.1
Sb	25.2	28.3
Te	54.8	58.6

## Data Availability

The data presented in this study are available on request from the corresponding author.

## Supplementary Materials

The following are available online at https://www.mdpi.com/1996-1944/14/3/536/s1, Figure S1. Line-scan profile in HR-TEM image of the as-synthesized BST NPs; Figure S2. EDS spectra of the as-synthesized BST NPs; Figure S3. SEM and TEM images of (a,b) as-synthesized BST NPs and (c,d) post wet-milled BST NPs, respectively; Figure S4. XRD patterns of as-synthesized BST NPs and post wet-milled BST NPs, respectively. Inset shows the full width at half maximum (FWHM) and crystallite size calculated from Scherrer equation.
