# Supplementary material for: Synthesis of Uniformly Sized Bi0.5Sb1.5Te3.0 Nanoparticles via Mechanochemical Process and Wet-Milling for Reduced Thermal Conductivity
Source: Materials (Basel). 2021 Jan 22;14(3):536. doi: 10.3390/ma14030536 (PMC7865327; doi:10.3390/ma14030536)
Supplement: Supplementary file 1 [file materials-14-00536-s001.pdf]

materials

supplementary

Synthesis of Uniformly Sized $Bi_{0.5}Sb_{1.5}Te_{3.0}$ Nanoparticles via Mechanochemical Process and Wet-Milling for Reduced Thermal Conductivity

Bo-In Park [1,2], Miri Shin [2], Jaeho Park [3], Jae-Seung Lee [4], Seung Yong Lee [2,*] and Seunggun Yu [5,*]

[1] Department of Materials Science and Engineering, Korea Advanced Institute of Science and Technology (KAIST), Daejeon 34141, Korea; boin0995@gmail.com
[2] Materials Architecturing Research Center, Korea Institute of Science and Technology (KIST), Seoul 02792, Korea; T20233@kist.re.kr
[3] Department of Materials Science and Engineering, Seoul National University, Seoul 08826, Korea; haha0728@kist.re.kr
[4] Department of Materials Science and Engineering, Korea University, Seoul 02841, Korea; jslee79@korea.ac.kr
[5] Insulation Materials Research Center, Korea Electrotechnology Research Institute (KERI), Changwon 51543, Korea
* Correspondence: patra@kist.re.kr (S.Y.L.); viola@keri.re.kr (S.Y.)

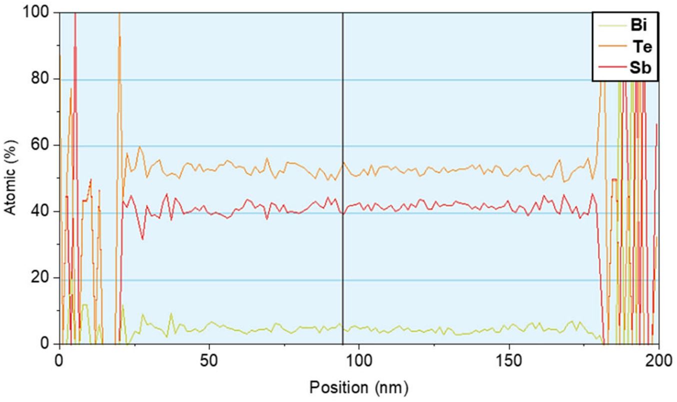

Figure 1. Line-scan profile in HR-TEM image of the as-synthesized BST NPs.

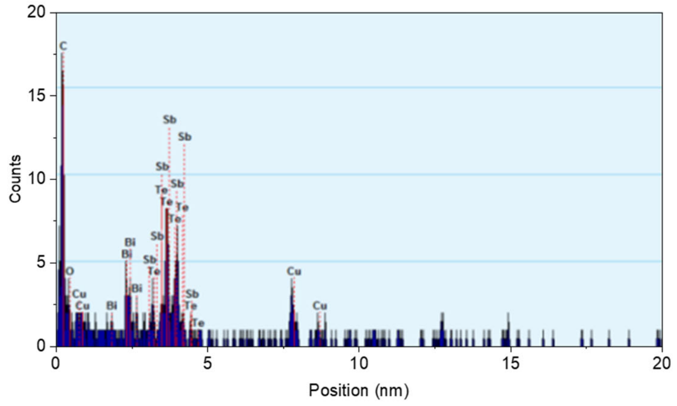

Figure 2. EDS spectra of the as-synthesized BST NPs.

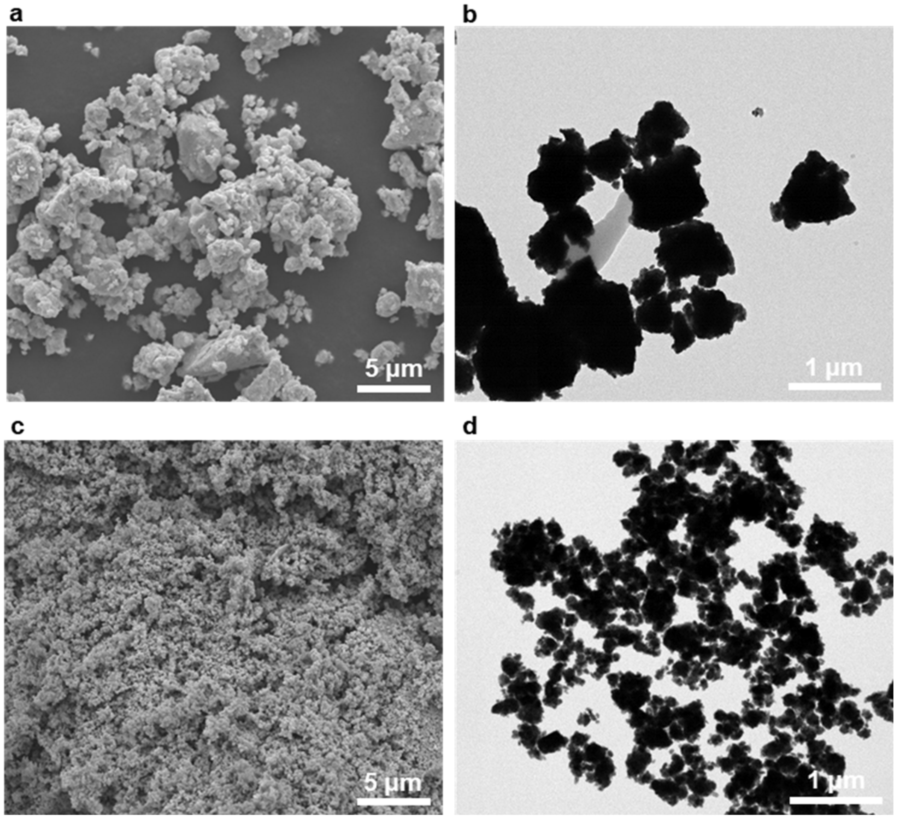

Figure 3. SEM and TEM images of (a,b) as-synthesized BST NPs and (c,d) post wet-milled BST NPs, respectively.

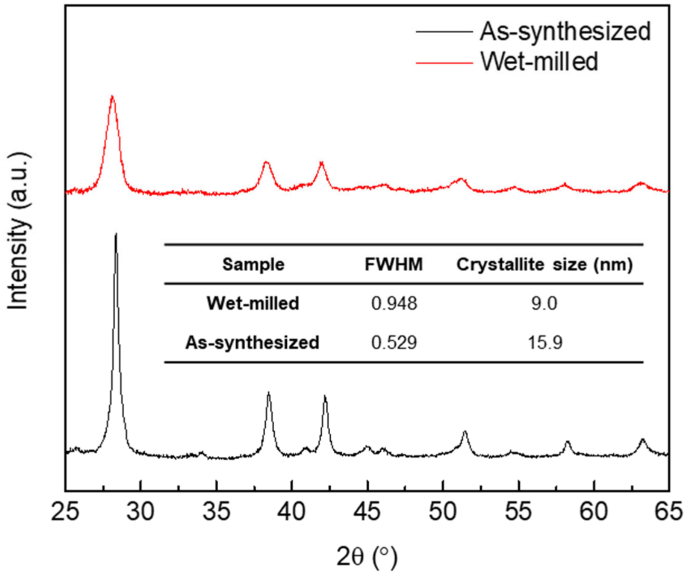

Figure 4. XRD patterns of as-synthesized BST NPs and post wet-milled BST NPs, respectively. Inset shows the full width at half maximum (FWHM) and crystallite size calculated from Scherrer equation.
